# Effects of High-Definition Transcranial Direct Current Stimulation (HD-tDCS) of the Intraparietal Sulcus and Dorsolateral Prefrontal Cortex on Working Memory and Divided Attention

**DOI:** 10.3389/fnint.2018.00064

**Published:** 2019-01-08

**Authors:** Stevan Nikolin, Shani Lauf, Colleen K. Loo, Donel Martin

**Affiliations:** School of Psychiatry, Prince of Wales Hospital, Black Dog Institute, University of New South Wales, Sydney, NSW, Australia

**Keywords:** divided attention, working memory, tDCS, HD-tDCS, IPS, DLPFC

## Abstract

**Objective:** There is a need to elucidate the underlying neural mechanisms subserving working memory and divided attention functioning. Recent neuroimaging studies provide evidence for anatomical co-localization of both functions. In the present study we used a functional intervention, whereby we applied a novel type of focalised, non-invasive brain stimulation, High-Definition transcranial Direct Current Stimulation (HD-tDCS), to the regions subserving these processes, the left intraparietal sulcus (IPS) and left dorsolateral prefrontal cortex (LDLPFC). Our aim was therefore to modulate activity in these regions using HD-tDCS and thereby assess their relevance for working memory, divided attention and their shared sub-processes.

**Method:** 78 participants were evenly randomized to one of three conditions in a single blind, parallel group study design. Anodal or sham HD-tDCS was applied to either the left IPS or LDLPFC while participants completed a verbal working memory task, a divided attention task, and two tasks measuring subcomponents of working memory (updating and maintenance).

**Results:** Focalised stimulation of the IPS and LDLPFC did not significantly modulate performance compared to sham stimulation. However, moderate effect sizes were obtained for at least one HD-tDCS condition relative to sham for all tasks, warranting further research into the functional importance of the IPS in subserving these abilities.

**Conclusions:** The current results may be useful for informing future tDCS studies for modulating working memory and divided attention functioning.

## Introduction

For some time, working memory and divided attention have been studied as two independent processes in cognitive neuroscience. However, recently, evidence has emerged which suggests an interdependency of these processes, both behaviourally and physiologically (de Fockert et al., 2001; McMains and Somers, 2005; Santangelo and Macaluso, 2013; Cowan et al., 2014). This research has suggested that both abilities share a common neural substrate involving bilateral regions of the fronto-parietal network, including the prefrontal cortex (PFC) and intraparietal sulcus (IPS). Neuroimaging evidence has suggested that the PFC may specifically play a role in updating during working memory (Kikyo et al., 2001; Nyberg et al., 2003), while the IPS instead functions to maintain representations for both working memory and divided attention (Santangelo and Macaluso, 2013). In the current study, we aimed to investigate the importance of these respective regions for modulating both working memory and divided attention performance and assess their hypothesized functions via administration of focalised brain stimulation.

Working memory refers to the short-term storage and processing of information from all sensory modalities. It has been proposed that working memory comprises three subcomponents: the central executive, the visuospatial sketch pad, which processes visual information, and the phonological loop, which stores and rehearses auditory information (Baddeley and Hitch, 1974; Baddeley, 1992). The central executive is believed to be responsible for the encoding and retrieval, or updating, of information in working memory (Morris and Jones, 1990), and the visuospatial sketch pad and phonological loop to be largely involved in the maintenance of information (Morris, 1987). Alternative theoretical frameworks have also been proposed which instead suggest a more unitary limited capacity construct (e.g., Cowan, 2001). Under this framework, stimuli from different modalities (e.g., auditory or visual) instead compete for a central focus of attention (Saults and Cowan, 2007), in contrast to being processed by specialized subsystems as in the aforementioned model. In this model, both updating and maintenance occurs in the central focus of the attention storage system, while storage or maintenance can also occur in separate peripheral storage for a single type of stimulus (Cowan et al., 2014).

Verbal working memory involves several regions which comprise a large-scale neural network (see Eriksson et al., 2015 for a comprehensive review). Selective attention and maintenance of temporal order rely on prefrontal regions (Wager and Smith, 2003; Hsieh et al., 2011). Maintenance of serial order across items in working memory has been anatomo-functionally correlated with the supramarginal gyrus in lesion studies (Papagno et al., 2017; Paulesu et al., 2017). Information manipulation activates the ventral frontal cortex and pre-supplementary motor areas (Wager and Smith, 2003; Nee et al., 2012). Semantic storage has been localized to temporal areas including the lateral temporal lobes and the temporoparietal cortex (Binder et al., 2009). Rehearsal of information to prevent verbal trace decay has been observed in the left inferior frontal gyrus (Paulesu et al., 1993) and, interestingly, in the cerebellum (Nee et al., 2012).

Functional neuroimaging has further provided evidence for different neural regions subserving the hypothesized components of working memory of updating and maintenance. Updating processes of working memory are subserved predominantly by areas within the PFC and particularly the dorsolateral region of the PFC (Cabeza and Nyberg, 2000). Specifically, activity within the left dorsolateral prefrontal cortex (LDLPFC) has been associated with the updating of information in verbal working memory (Kikyo et al., 2001; Nyberg et al., 2003). In contrast, for maintenance functions, activation of the posterior parietal cortex, and specifically the IPS has been implicated (Jha and McCarthy, 2006; Santangelo and Macaluso, 2013). For verbal maintenance tasks, the left IPS is recruited (Cabeza and Nyberg, 2000). Other studies, however, have suggested that the IPS may also be involved in additional functions, including manipulation processes (Champod and Petrides, 2007), or both maintenance and manipulation (Gazzaley et al., 2004; Bray et al., 2013). While the causal roles of these brain regions for modulating working memory performance have previously been examined using non-invasive brain stimulation (i.e., LDPFC: Mottaghy et al., 2000; Osaka et al., 2007; Brunoni and Vanderhasselt, 2014; IPS: Oliveri et al., 2001; Luber et al., 2007; Hamidi et al., 2008; Tseng et al., 2010), specific effects on these respective component sub-functions have yet to be investigated.

Divided attention involves deploying attention to multiple sensory stimuli simultaneously, and similarly to working memory is critical to the performance of complex daily activities, for example, driving (Devos et al., 2014). Neuroimaging studies have found activation of the DLPFC and IPS during divided attention performance, thereby implicating a shared neural substrate with working memory. Fagioli and Macaluso (2009) used fMRI to show that visuospatial divided attention activates a fronto-parietal network, including the IPS and DLPFC. A common dorsal frontoparietal attention network was further shown to be involved in monitoring both single and dual modalities, with increased activity during the dual monitoring condition (Santangelo et al., 2010). Santangelo and Macaluso (2013) investigated the effect of increasing working memory load on activity associated with divided attention performance and found that this was correlated with increased activity in the IPS. More recently, Santangelo (2018) showed that divided attention tasks involve recruitment of nodes from both the dorsal frontoparietal and salience networks, thereby suggesting recruitment of large scale networks during performance. Based on these findings, it has been hypothesized that both divided attention and working memory may share a common pool of processing resources (Santangelo and Macaluso, 2013). However, this relationship has yet to be causally demonstrated.

Transcranial direct current stimulation (tDCS), a mild non-invasive brain stimulation technique, has previously been utilized as a tool to enhance cognitive functioning via the modulation of cortical activity tDCS involves the passing of very weak electric currents through electrodes placed on the scalp, to modulate the resting potentials of underlying neural tissue. It has been successfully employed to show the functional importance of stimulated brain regions across multiple cognitive functions (Coffman et al., 2014). However, a feature of conventional tDCS is diffuse cortical stimulation, whereby the alterations in cortical excitability may spread to other areas external to the targeted region (Nathan et al., 1993; Bai et al., 2014). Functional neuroimaging studies of conventional tDCS indeed have shown stimulation induced changes in non-targeted distant regions (Keeser et al., 2011; Stagg et al., 2013). Romero Lauro et al. (2014) probed the cortical excitability effects of tDCS delivered over the posterior parietal cortex using transcranial magnetic stimulation and found increased excitability in nearby parietal regions as well distal bilateral frontal regions. TDCS preferentially enhances excitability in regions within active large-scale neural networks, demonstrating neurophysiological functional specificity despite stimulation of a large proportion of the brain (Pisoni et al., 2018). This presents difficulties for interpreting functioning for specific cortical regions.

High Definition transcranial Direct Current Stimulation (HD-tDCS) is a newly developed form of tDCS, that utilizes smaller electrodes, and electrode montages that proficiently limit the spread of current flow outside of the target area, as suggested by computer modeling (Datta et al., 2009; Kuo et al., 2013) and studies of motor cortex excitability (Edwards et al., 2013). HD-tDCS may therefore provide a more focalised stimulation that lends itself well to specifically modulating activity only within the region of interest. HD-tDCS has been used to enhance various cognitive functions including response inhibition and naming accuracy (Richardson et al., 2015; Gbadeyan et al., 2016). We recently successfully employed HD-tDCS to probe different cortical regions involved in verbal learning, showing directly that the LDLPFC is important for working memory performance (Nikolin et al., 2015). To the best of our knowledge, HD-tDCS has not yet been used to examine the functional specificity of cortical regions subserving working memory and divided attention, or their relevant sub-processes.

In the current study, we therefore aimed to directly investigate the functional importance of the LDLPFC and left IPS for both working memory and divided attention performance using HD-tDCS. Our secondary aim was to investigate the roles of the LDLPFC and left IPS in subserving functions considered important for both working memory and divided attention performance, namely, updating and maintenance. We hypothesized that focalised stimulation of both the LDLPFC and left IPS would upregulate activity within these regions and improve both working memory and divided attention performance compared to sham, and that stimulation of the LDLPFC would specifically improve updating performance, whilst stimulation of the left IPS would improve maintenance capacity.

## Materials and Methods

### Design

The study used a single blind, parallel group, sham-controlled experimental design. Participants were randomized into one of three groups: Group 1 (active LDLPFC HD-tDCS), Group 2 (active left IPS HD-tDCS) and Group 3 (sham HD-tDCS). This design enabled the elimination of carry-over effects of stimulation and practice effects on the cognitive tasks. Participants were blinded to their stimulation condition.

### Participants

A power analysis was conducted using an effect size of Cohen's *d* = 0.80 obtained from a previous study conducted by our group that examined anodal tDCS of the LDLPFC on a divided attention task in healthy subjects (Martin et al., 2013). It was estimated that a sample size of 78 participants (26 per group) would be sufficient to detect a significant difference between active and sham conditions on the divided attention task used in this study (power = 80%, alpha = 0.05, two-tailed). Participants were recruited from the University of New South Wales (age 22.2 years ± 4.0; 51 female) through an advertisement on the careers website, and the School of Psychology first year study participation scheme. Inclusion criteria were: aged between 18 and 40, right-handed assessed by the Edinburgh Handedness Inventory (Oldfield, 1971), fluent in English, not taking any concurrent medications which may affect cognitive performance, free from any neurological or psychiatric disorder, no recent head injury, no history of seizure or stroke, and no current history of drug or alcohol abuse or dependence. This study was approved by the human research ethics committee of the University of New South Wales, and performed in accordance with the principles outlined in the Australian National Statement of Ethical Conduct in Human Research. Written and informed consent was obtained from all participants prior to commencing the study.

### Study Protocol

**Figure 1:**
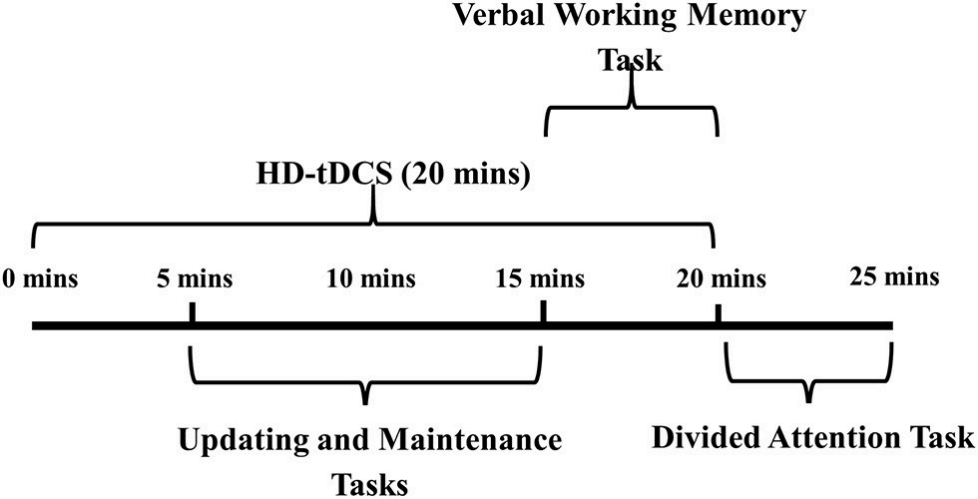
Testing session procedure.HD-tDCS, high definition transcranial direct current stimulation.

All participants completed the same cognitive tasks in a one-off testing session. The tasks consisted of an updating task, a maintenance task, a verbal working memory task and a divided attention task. Prior to the session, participants practiced each of the tasks for two trials. Participants then received 20 min of HD-tDCS (or sham HD-tDCS), 5 min of which was delivered prior to the start of cognitive testing (Figure 1 shows the schedule for the testing session). Participants commenced the updating task and the maintenance task, which were presented in a randomized and counterbalanced order. Next, participants completed a verbal working memory task (consisting of only auditory components of the three-back task), and then a divided attention task (consisting of both auditory and visual components of the three-back task).

### High-Definition Transcranial Direct Current Stimulation (HD-tDCS)

**Figure 2:**
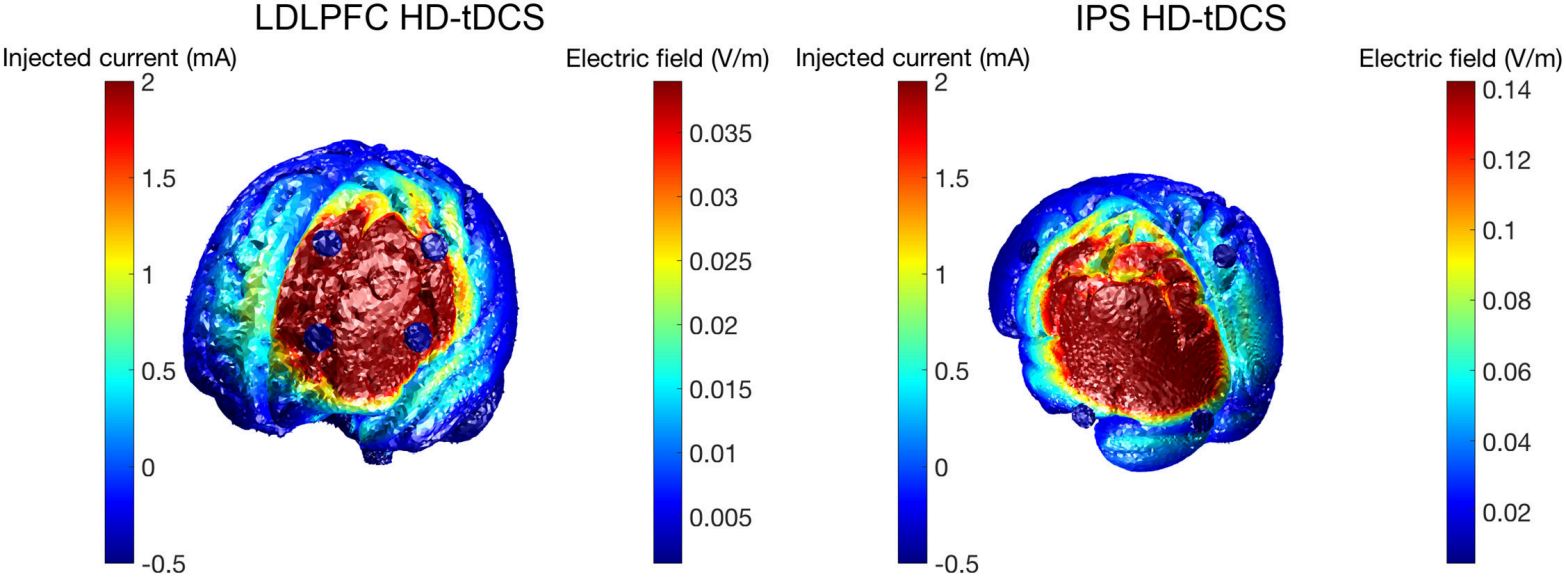
Electric field magnitude (V/m) for HD-tDCS conditions.

**Figure 3:**
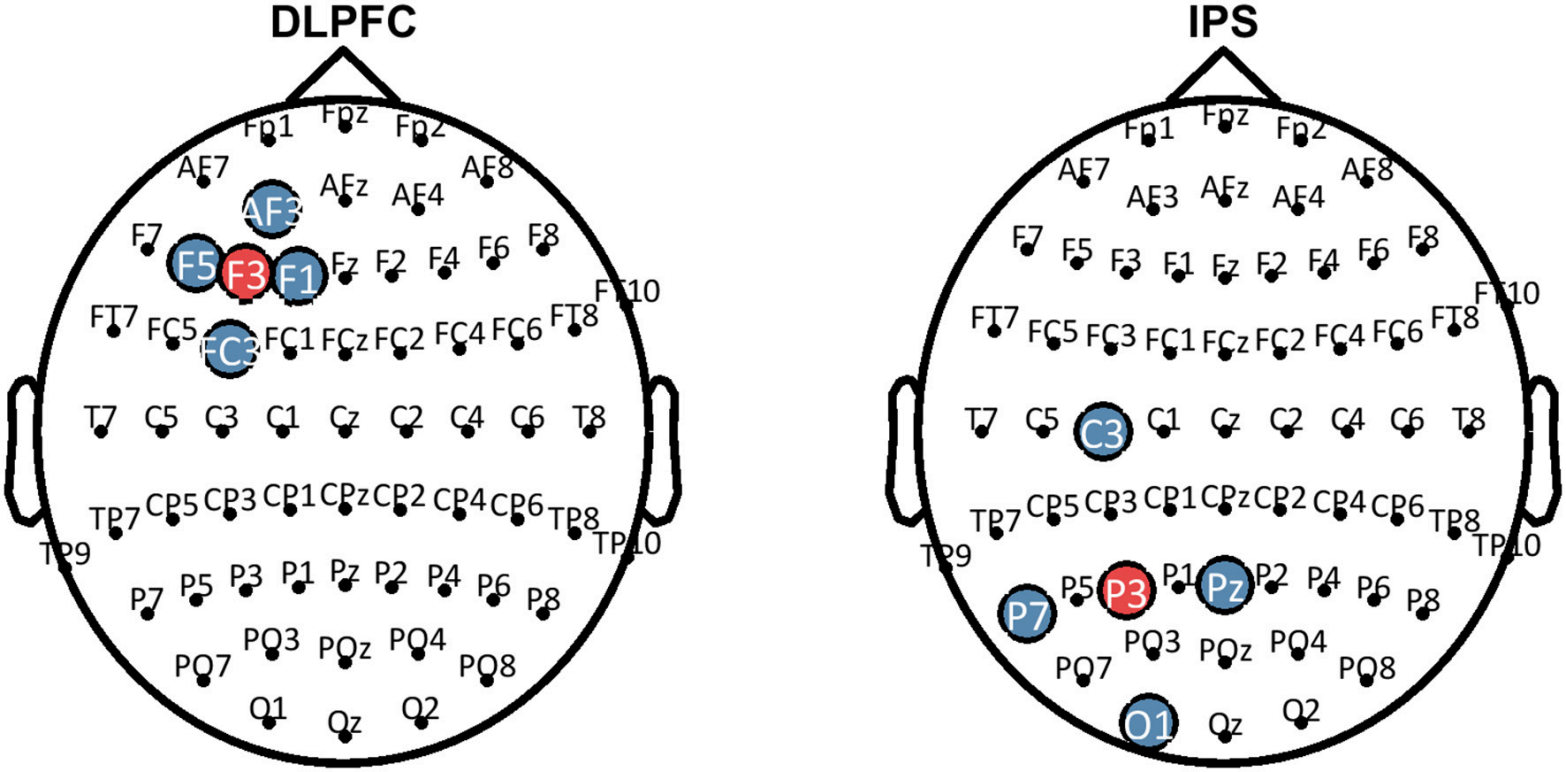
Montages that were used for HD-tDCS. Blue sites represent the cathodes, red sites represent the anodes. HD-tDCS, high-definition transcranial direct current stimulation; LDLPFC, left dorsolateral prefrontal cortex; IPS, intraparietal sulcus.

HD-tDCS (Soterix Medical, New York, NY, United States) was delivered for 20 min continuously in both Groups 1 and 2, at a current intensity of 2 mA. Group 1 received anodal stimulation of the LDLPFC (F3 according to the international 10–20 EEG system) using a 4 x 1 ring electrode configuration whereby cathodes were placed on adjacent electrode sites (F5, AF3, F1, FC3). Group 2 received anodal stimulation of the left IPS (P3) in the same 4 x 1 electrode configuration with cathodes placed at P7, Pz, C3, and O1. Figure 2 shows computational modeling results of the electric field distribution for IPS and LDLPFC HD-tDCS montages created using the open source tDCS modeling package Realistic vOlumetric-Approach to Simulate Transcranial Electric Stimulation (ROAST; for more details see Huang et al., 2017). Cathodes were spaced further apart for left IPS stimulation, as compared to the LDLPFC montage, to produce an equivalent electric field strength in the sulcus at depth as suggested by computational modeling software (Soterix Medical, New York, NY, United States). Electrode sizes were 3.14 cm^2^. Figure 3 shows the montages used. Due to the proximity of the cathode at C3 to the motor cortex, participants in all groups were asked to respond on tasks measuring reaction time using their left hand. Current in these two conditions was ramped up over 30 s, and gradually back down over 30 s at the cessation of stimulation.

Participants in the sham condition were randomized to either the left IPS montage or the LDLPFC montage, and electrodes were placed in the same montages as in the active groups. The current in the sham condition was ramped up to 1 mA over 30 s, and then back down over another 30 s and switched off. The HD-tDCS machine was placed behind participants and all adjustments to the current in all conditions were made out of view to the participants to preserve blinding.

### Cognitive Tasks

Cognitive tasks were all administered via computer using a custom Inquisit script (Version 4, Millisecond Software LLC, Seattle, WA, United States). Auditory tasks were administered through headphones. Tasks were selected based on past literature demonstrating that they predominantly assessed one facet of cognitive functioning (e.g., divided attention). However, it should be noted that performance on all cognitive tasks is dependent to some extent on synergistic functioning between multiple cognitive systems and sub-processes. The exact degree of interdepedency between cognitive systems, notably divided attention and working memory sub-processes, could not be assessed using the present study design due to sequential task presentation. Nevertheless, this study design allowed us to examine the functional relevance of the LDLPFC and IPS for the cognitive functions being assessed.

#### Verbal Working Memory Task

The verbal working memory task consisted of an auditory 3-back task adapted from Jaeggi et al. (2008). This task was chosen as a general measure of working memory performance, based on its ability to activate a fronto-parietal network including the LDLPFC (Smith and Jonides, 1997; Courtney et al., 1998; Nystrom et al., 2000; Hautzel et al., 2002) and the posterior parietal cortex (Owen et al., 2005). Participants heard a series of eight different letters presented one a time, with each letter presentation lasting 1,000 ms followed by an interstimulus interval lasting 1,000 ms. Participants were asked to respond using the spacebar whenever the current letter matched a target letter presented three items back. The three-back task consisted of 24 targets (correct possible responses) among 108 cues [non-targets). D-prime (calculated as z(hit rate)-z(false alarm rate)] was calculated to provide a measure of verbal working memory accuracy. Reaction times (RTs) of correct responses were also recorded as a secondary outcome measure. Overall duration of the verbal working memory task was approximately 5 min.

#### Divided Attention Task

The divided attention task consisted of both auditory and visual components of the dual three-back task adapted from Jaeggi et al. (2008). Participants heard a series of eight different letters presented one at a time, each paired simultaneously with a visual stimulus. The visual stimulus consisted of a box presented in one of eight locations around the periphery of the screen. Each paired stimuli presentation lasted 500 ms, followed by an interstimulus interval lasted 2,500 ms, during which participants could respond. Participants were asked to respond by pressing the “A” key whenever the current location of the box matched the location three trials back, and by pressing the “L” key whenever the current letter matched the letter participants heard three items back. Thus, participants were required to monitor both auditory and visual information simultaneously. The dual three-back task consisted of 16 auditory targets, 16 visual targets, eight combined targets (visual and auditory target presented simultaneously) and 40 non-targets. D-prime [calculated as z(hit rate)–z(false alarm rate)] was calculated to provide a measure of divided attention accuracy. Overall duration of the divided attention task was approximately 5 min.

#### Updating Task

The updating task consisted of a running letter count adapted from Lilienthal et al. (2013). Participants heard a series of letters, C and D, one at a time, in a randomized order. With each presentation, participants were required to update their count of how many of each letter they had heard so far in the series, and then to press spacebar to hear the next letter. As such two running counts were required to be kept; one for each letter. Participants were asked to report both counts at the end of each series by typing responses into the computer. RTs were recorded from the presentation of the letter to when participants pressed the spacebar, between repeat trials (i.e., C-C, D-D) as this has been shown to measure verbal updating performance (Lilienthal et al., 2013). The RT therefore reflects the average time taken for the participants to update their letter count and proceed to the next letter, without incorporating the slowing that occurs from an attentional switch to a dissimilar letter (e.g., C–D). Series of letters were presented in lengths of 16 and 20 letters, with two trials of each length (a total of four trials). The outcome measure for this task was RTs between repeat trials. The overall duration of the updating task was approximately 4 min.

#### Maintenance Task

The maintenance task consisted of an operation span task (Turner and Engle, 1989). Span tasks have often been used as measures of maintenance (Cabeza and Nyberg, 2000). The operation span task was modified to include an interference component such that maintenance is more accurately tested in its most pressed capacity (see Conway et al. (2005) for a review). Participants saw a series of letters presented, during which they were required to perform simple maths calculations (in between each letter presentation, shown on the screen). At the end of the trial, participants were asked to recall as many letters as possible from the beginning of the series, in order of presentation. Letters were presented in series lengths of 4, 6, 8, and 10 letters in a random order, with two trials of each length (making a total of eight trials). One point was awarded for each letter recalled in the correct serial position. The outcome measure of this task was the total number of letters recalled for all levels of difficulty (series length) combined, representing verbal maintenance capacity. Total duration for the maintenance task was approximately 6 min.

### Statistical Analysis

All statistical analyses were conducted using the Statistical Package for the Social Sciences (SPSS) for Windows, Version 22 (IBM corp., Armonk, NY). Firstly, one-way analysis of variances (ANOVAs) were conducted to examine group differences for the demographic variables age and years of education. Chi-Square tests were also conducted to detect differences between conditions for gender and blinding. Demographic variables were included as covariates in the analysis of all outcome measures in the event of significance. Reaction time data was cleaned prior to analysis, with all responses < 250 ms being removed from the analysis. Participants' results were excluded from the analysis of outcome measures if they performed outside of the range of three standard deviations away from the study mean for that outcome. ANCOVAs were then conducted to test for main effects of condition on all outcome measures. Greenhouse-Geisser corrections were applied if there were violations of Mauchley's test of sphericity. Statistical significance was set using an alpha level < 0.05.

Additionally, Bayesian statistics were calculated using the software package Jeffreys's Amazing Statistics Program (JASP) (Version 0.8.6, JASP Team). Bayesian ANCOVAs were conducted to evaluate the relative strength of evidence in favor of the alternative hypothesis (H_1_) compared to the null hypothesis (H_0_). Bayes factors wer used to quantify the degree to which the data favor H_1_ relative to H_0_ (BF_10_). A BF_10_ >3 indicates substantial evidence for H_1_ (Wagenmakers et al., 2018a,b). A BF_10_ between 1/3 and 3 indicates data insensitivity in distinguishing H_0_ and H_1_. Finally, a BF_10_ < 1/3 (i.e., < 0.33) indicates substantial support for the null hypothesis (Dienes, 2014).

## Results

### Demographics

Analysis of demographic differences between conditions revealed significant differences between conditions in age and years of education (i.e., *p* = 0.02 and *p* = 0.03, respectively). Given that years of education is correlated with age in this university sample (*r* = 0.645, *p* < 0.001), subsequent analyses only included age as a covariate to control for the differences between groups. There were no significant differences observed for gender between groups. See Table 1 for details of demographics for each condition.

**Table 1:** Demographic data.

	**Group, means (SEM)**				
	**Sham**	**LDLPFC**	**Left IPS**	***F*/χ^2^**	***p***
*n*	26	26	26	–	–
Age	20.46 (0.49)^a^	22.58 (0.94)	23.50 (0.76)^a^	4.27	0.02
Years of education	13.81 (0.43)^a^	14.50 (0.46)	15.54 (0.47)^a^	3.67	0.03
Female/Male	16/10	18/8	17/9	0.34	0.84

*SEM, standard error of the mean; LDLPFC, left dorsolateral intraparietal sulcus; IPS, intraparietal sulcus*.

a *Difference in age and years of education was significant between left IPS and sham conditions, p < 0.05*.

### Verbal Working Memory

Table 2 shows the results for verbal working memory. There was no significant main effect of stimulation condition for either d-prime [*F*_(2, 74)_ = 1.44, *p* = 0.24, η^2^ = 0.04] or RT for hits [*F*_(2, 74)_ = 1.03, *p* = 0.36, η^2^ = 0.03]. Bayesian ANCOVAs found substantial support for the null hypothesis for both d-prime (BF_10_ = 0.08) and RT (BF_10_ = 0.06).

**Table 2:** Results for the updating, working memory, divided attention, and maintenance tasks.

	**Group, means (SEM)**					**Effect size (Cohen's** ***d)***	
**Measure**	**Sham**	**LDLPFC**	**Left IPS**	***F* condition**	***p***	**Sham *vs*. LDLPFC**	**Sham *vs*. IPS**
Updating RT (ms)	1190.48 (42.65)	1269.34 (57.14)	1191.75 (48.75)	0.78	0.46	0.31	0.01
3-back d-prime	1.53 (0.10)	1.53 (0.11)	1.75 (0.12)	1.44	0.24	0.14	0.38
3-back RT hits (ms)	974 (29.5)	976 (28.6)	1027 (29.2)	1.03	0.36	0.01	0.35
Dual 3-back d-prime	0.93 (0.07)	1.11 (0.09)	1.20 (0.09)	2.91	0.06	0.42	0.63
Operation span task	29.48 (1.79)	29.92 (1.62)	33.04 (1.55)	1.80	0.17	0.05	0.43

*Age was included as a covariate in the analysis of all outcome measures. SEM, standard error of the mean; RT, reaction time; LDLPFC, left dorsolateral prefrontal cortex; IPS, intraparietal sulcus; CI, confidence interval*.

### Divided Attention

**Figure 4:**
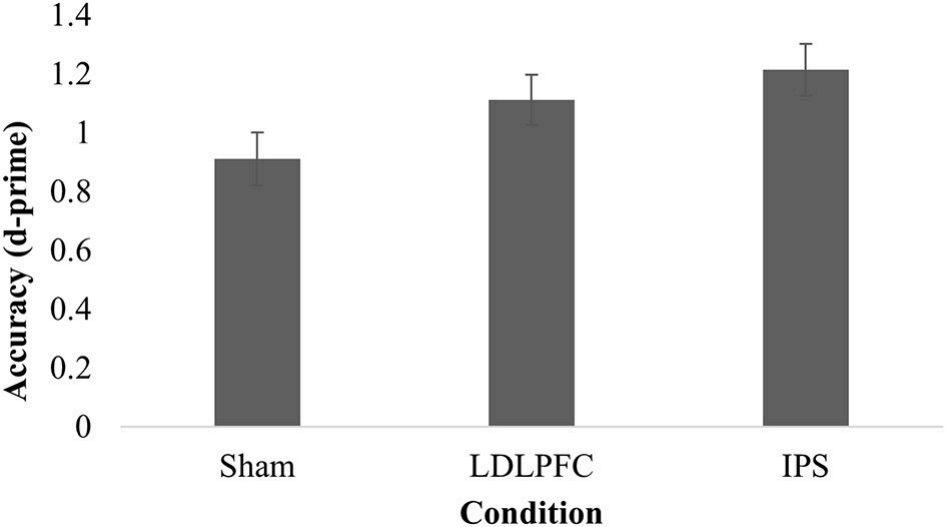
Divided attention. Mean discriminative sensitivity on the divided attention task for each stimulation condition. LDLPFC, left dorsolateral prefrontal cortex; IPS, intraparietal sulcus.

Table 2 shows the results for divided attention. The results for one participant were excluded from analysis as an outlier (1.3% of the total sample), due to being more than three SD saway from the mean. The main effect of stimulation condition for d-prime was not significant [*F*_(2, 73)_ = 2.91, *p* = 0.06, η^2^ = 0.07]. A Bayesian ANCOVA found substantial support for the null hypothesis (BF_10_ = 0.23). Figure 4 shows the results for the divided attention outcome measure.

### Updating

Table 2 shows the results for the updating task. The results for one participant were excluded from analysis in this task due to not following instructions (1.3% of the total sample). There was no significant main effect of stimulation condition for reaction time between repeat trials on the running letter count task [*F*_(2, 73)_ = 0.78, *p* = 0.46, η^2^ = 0.02]. A Bayesian ANCOVA found substantial support for the null hypothesis (BF_10_ = 0.10).

### Maintenance

**Figure 5:**
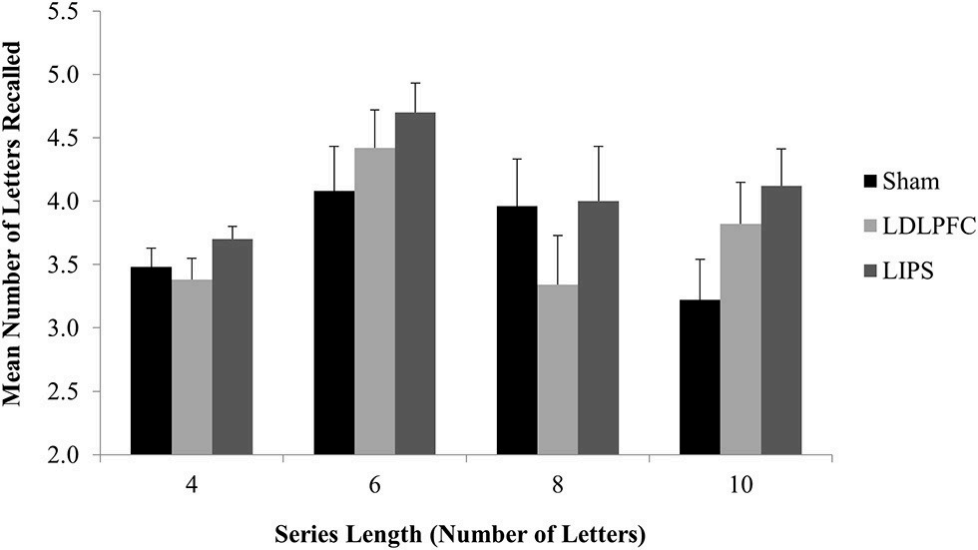
Maintenance Task. Mean number of letters recalled for each series length, by stimulation condition. LDLPFC, left dorsolateral prefrontal cortex; LIPS, left intraparietal sulcus.

The results for three participants (3.8% of the total sample) were excluded from analysis as outliers (more than three SDs away from the mean). There was no significant main effect of stimulation condition for total number of letters recalled [*F*_(2, 71)_ = 1.80, *p* = 0.17, η^2^ = 0.05]; Table 2. A Bayesian ANCOVA found substantial support for the null hypothesis (BF_10_ = 0.11). Figure 5 shows the results for the maintenance outcome measure.

### Assessment of Blinding

Blinding was assessed in 69/78 participants at the end of the session. Participants were asked to guess which condition they had been allocated to in a forced selection between active or sham stimulation, regardless of their level of uncertainty. There were no differences in participant guesses between conditions [χ^2^ = 1.32 (2), *p* = 0.52]. In the IPS condition 6/22 guessed active, LDLPFC condition 7/23 guessed active and sham condition 4/20 guessed active.

### Adverse Events

All but two participants tolerated the stimulation well. These participants reported sharp stinging and burning sensations at the site of the electrode, prior to the maximal current level being reached, and as such stimulation could not be continued and they were excluded from the study. All other participants reported much milder side effects, consisting of tingling, itching, mild burning and mild stinging, that typically lasted between 2 and 10 min (see Supplementary Table 1). There was no difference in adverse event frequency between conditions with the exception of pain, which was reported more often in LDLPFC HD-tDCS compared to sham and IPS HD-tDCS (Pearson's χ^2^ = 7.58, *p* = 0.02).

## Discussion

Recent neuroimaging research has suggested that both working memory and divided attention abilities share common neural substrates. The aim of this study was to directly investigate the specific roles of the LDLPFC and IPS in modulating performance for both abilities through administration of HD-tDCS, a focalised non-invasive brain stimulation method. A secondary aim was to further investigate the roles of these regions in supporting cognitive functions hypothesized to be important for performance of both abilities, namely updating and maintenance.

To achieve these aims, HD-tDCS was used to directly modulate activity in these key regions (IPS and LDLPFC), allowing causal inference of their functional roles in the previously mentioned cognitive processes. Past research has successfully applied HD-tDCS in this manner to probe cognition (Richardson et al., 2015), including within the domain of working memory (Nikolin et al., 2015). Results from the present study, however, did not identify statistically significant differences between the control condition and HD-tDCS conditions for any of the examined cognitive measures. It is important to note that failure to reject the null hypothesis, as was the case in the present study, does not equate to the null hypothesis being true i.e., that there is no difference in cognitive performance between conditions. An equivalence test would be required to demonstrate the absence of an effect (Lakens, 2017). Indeed, further examination of effect sizes (Cohen's d) reveals a small-moderate effect for each cognitive test for at least one HD-tDCS condition as compared to sham stimulation, similar to the effect sizes observed in the tDCS literature (Hill et al., 2016). Effect sizes gauge the difference between conditions and can be used to provide an estimate of substantive significance (Nakagawa and Cuthill, 2007; Sullivan and Feinn, 2012). Interestingly, IPS HD-tDCS effect sizes relative to the sham condition were numerically greater than effect sizes for LDLFPC HD-tDCS for divided attention and maintenance tasks, but not for updating processes. It is possible that these differences in effect sizes could be due to greater spacing between electrodes for IPS HD-tDCS relative to the LDPFC HD-tDCS electrode montage (see Figure 2). Alam et al. (2016) modeled the effects of increased ring electrode radius, noting both an increase in the depth of current penetration as well as an increase in the peak electric field. Increased distance between the anode and cathode electrodes reduces shunting of electrical current across the skin, activating a larger area of the brain, therefore allowing greater relative modulation of the fronto-parietal working memory network (Culham and Kanwisher, 2001).

Prior neuroimaging work has provided correlational evidence of the role of the IPS in supporting divided attention functioning. Fagioli and Macaluso (2009), for example, found that performance during visuospatial divided attention consistently activated a fronto-parietal network, comprising subcomponents including the DLPFC and the IPS, using fMRI. Similarly, Santangelo and Macaluso (2013) examined neural activation patterns while participants monitored multiple object categories or locations and found activation of the dorsal fronto-parietal network, including the IPS. Interestingly, when load on the task was increased, activation patterns further intensified in the area of the IPS bilaterally, thereby further implicating a role of the IPS in subserving performance. The superior parietal lobule, an area bordered by the IPS, has also been directly implicated as a node within the dorsal attention network (Spreng et al., 2013), and may thus also exert functional control over brain regions relevant to attentional processing. Modulation of IPS activity using non-invasive brain stimulation has further provided some causal evidence for its relevance to divided attention. Excitatory anodal stimulation using standard tDCS was administered to area C4, corresponding to a region between the right TPJ and right IPS, with the cathode placed over the contralateral upper arm, improved performance on a visuospatial divided attention training task (Scheldrup et al., 2014). However, due to the wide spacing of the electrodes in that study, which thus resulted in generalized stimulation of the dorsal attention network, the functional role of the IPS alone in facilitating performance cannot be determined. Despite substantial effect sizes for IPS and LDLPFC HD-tDCS conditions relative to sham stimulation, significance testing revealed no statistically meaningful difference between conditions, and as such this study is unable to validate this interpretation.

That we did not observe modulation of either updating or working memory performance with focalised stimulation of the LDLPFC is of interest. Multiple prior neuroimaging studies have implicated a role of the LDLPFC in verbal updating (Petrides, 1994, 2000; Owen, 1997; D'Esposito et al., 1999; Kikyo et al., 2001; Nyberg et al., 2003; Barbey et al., 2013). A possible reason for the lack of an effect could be that updating processes may require the involvement of a network of structures, beyond just the LDLPFC. The current results also contrast with our prior study using LDLPFC HD-tDCS which showed improved reaction times on a working memory task using a cross over study design (Nikolin et al., 2015). It is possible that this latter null finding may then be due to insufficient power in the current study which used a parallel group design. Alternatively, it is possible that the use of multiple mentally taxing cognitive tasks in this study may have fatigued participants and limited the capacity for tDCS to improve performance, particularly on the later more complex tasks (e.g., which assessed working memory and divided attention). Nevertheless, this study is the largest trial to-date using HD-tDCS to investigate cognitive processes, with a total sample of 78 participants, and was adequately powered on the basis of existing literature at the time the protocol was conceptualized. Subsequently, a recent meta-analysis showed an overall small sized positive effect (Hedge's g = 0.10) of “standard” anodal LDLPFC tDCS in reducing reaction times, but not accuracy, in healthy participants using cross-over study designs (Dedoncker et al., 2016). Cross-over study designs have more power to detect small effects due to reduced inter-individual variability, which has been identified as an important factor for consideration with non-invasive brain stimulation (López-Alonso et al., 2014; Chew et al., 2015).

There were several limitations to this study. Firstly, the montages utilized in this study were based on theoretical computer modeling, and therefore do not account for inter-individual neuroanatomical variability. As such, it is possible that the cortical target of interest was not optimally stimulated in all participants. Future HD-tDCS studies could incorporate neuroimaging and/or neuronavigational software to more precisely target regions of interest. Secondly, cognitive tasks were not all administered in a counterbalanced and randomized order and consequently could have been prone to order effects. We note, however, that the reason for opting not to deliver them in this manner was due to the difficult nature of some of the tasks. Specifically, we chose to administer the most difficult task (divided attention) last to minimize fatigue during the experiment. Additionally, baseline cognitive performance was not assessed. As such, it was not possible to examine interactions between inter-individual differences in memory capacity and the cognitive effects of HD-tDCS. Lastly, computer modeling and motor cortex studies suggest that the electric fields generated by HD-tDCS are restricted to the area bounded by the outer ring of electrodes (Edwards et al., 2013; Alam et al., 2016). However, further research is required to confirm that the modulatory effects of HD-tDCS are restricted to brain regions beneath the central electrode, rather than direct effects on functionally connected cortical/sub-cortical areas.

## Conclusions

No statistically significant effects were observed following LDLPFC and left IPS stimulation on any outcome measures. Moderate effect sizes were observed on working memory, divided attention, updating and maintenance tasks in at least one of the HD-tDCS conditions. Lack of significance may therefore be indicative of significant inter-individual variability in response. Further investigations of HD-tDCS as a neuropsychological probe are needed using larger sample sizes and cross over study designs to increase statistical power to detect differences between conditions.

## Ethics Statement

This study was carried out in accordance with the recommendations of the National Statement on Ethical Conduct in Human Research, with written informed consent from all subjects. All subjects gave written informed consent in accordance with the Declaration of Helsinki. The protocol was approved by the human research ethics committee of the University of New South Wales (HC15191).

## Author Contributions

SN, SL, CL, and DM: contributed conception and design of the study; SL: organized the database; SL and DM: performed the statistical analyses; SL: wrote the first draft of the manuscript; SN, SL, CL, and DM: wrote sections of the manuscript. All authors contributed to manuscript revision, read and approved the submitted version.

### Conflict of Interest Statement

The authors declare that the research was conducted in the absence of any commercial or financial relationships that could be construed as a potential conflict of interest.

## Abbreviations

tDCS: transcranial direct current stimulation
HD-tDCS: high-definition transcranial direct current stimulation
IPS: intraparietal sulcus
LDLPFC: left dorsolateral prefrontal cortex
PFC: prefrontal cortex.
