# Supplementary material for: Effects of High-Definition Transcranial Direct Current Stimulation (HD-tDCS) of the Intraparietal Sulcus and Dorsolateral Prefrontal Cortex on Working Memory and Divided Attention
Source: Front Integr Neurosci. 2019 Jan 8;12:64. doi: 10.3389/fnint.2018.00064 (PMC6331442; doi:10.3389/fnint.2018.00064)
Supplement: Supplementary file 1 [file Data_Sheet_1.docx]

**Supplementary Materials**

**Supplementary Table 1.** Number of participants that experienced an adverse event in each condition. Events are sorted according to overall likelihood of occurrence, with events most likely to occur listed first. Significance of adverse event occurrence was tested using Pearson Chi-Square tests. Pain was more likely to occur in the LDLPFC HD-tDCS condition compared to both sham and IPS HD-tDCS (*p* = 0.02).

| **Adverse event** | **Sham** | **LDLPFC** | **IPS** | **χ2** | **p-value** |
| --- | --- | --- | --- | --- | --- |
| Tingling | 8 | 13 | 13 | 2.607 | 0.272 |
| Burning | 8 | 11 | 11 | 0.975 | 0.614 |
| Itching | 5 | 4 | 8 | 1.956 | 0.376 |
| Pain | 0 | 5 | 1 | 7.583 | 0.023 |
| Dizziness | 1 | 0 | 0 | - | - |
| Headache | 0 | 0 | 1 | - | - |
| Fatigue | 1 | 0 | 0 | - | - |
